# Tumor microenvironment remodeling across thyroid cancer differentiation states revealed by spatial transcriptomics

**DOI:** 10.1007/s00262-025-04210-0

**Published:** 2025-11-03

**Authors:** Jungirl Seok, Hongyoon Choi, Eun Kyung Lee, Chang Hwan Ryu, Dongjoo Lee, Junsun Ryu, Seog Yun Park, Yuh-Seog Jung

**Affiliations:** 1 Department of Otorhinolaryngology-Head and Neck Surgery, National Cancer Center, 323 Ilsan-ro, Ilsandong-gu, Goyang-si, Gyeonggi-do 10408 Republic of Korea; 2 Department of Otorhinolaryngology-Head and Neck Surgery, Seoul National University Hospital, Seoul National University College of Medicine, Seoul, Republic of Korea; 3 Department of Nuclear Medicine, Seoul National University Hospital, Seoul National University College of Medicine, Seoul, Republic of Korea; 4 Portrai, Inc., Seoul, Republic of Korea; 5 Department of Internal Medicine, National Cancer Center, Goyang, Republic of Korea; 6 Department of Pathology, National Cancer Center, 323 Ilsan-ro, Ilsandong-gu, Goyang-si, Gyeonggi-do 10408 Republic of Korea

**Keywords:** Tumor microenvironment, Thyroid differentiation score, Spatial transcriptomics, JAK-STAT pathway, VEGF pathway

## Abstract

**Background:**

Thyroid cancer is the most common endocrine malignancy, with an increasing incidence, particularly driven by differentiated thyroid cancers (DTC), including papillary thyroid carcinoma (PTC) and follicular thyroid carcinoma (FTC). Although DTC generally have excellent prognosis, dedifferentiated forms such as poorly differentiated thyroid cancer (PDTC) and anaplastic thyroid cancer (ATC) exhibit aggressive progression and poor outcomes. Understanding the tumor microenvironment (TME) is crucial for developing novel therapeutic strategies.

**Methods:**

We performed spatial transcriptomics (ST) on formalin-fixed paraffin-embedded (FFPE) tumor tissues from 12 patients with PTC, FTC, PDTC, or ATC using the Visium CytAssist platform. Cell type composition was inferred using the CellDART algorithm, and pathway activity was assessed via PROGENy and REACTOME analyses. Immunohistochemical (IHC) assays were conducted to validate key TME components.

**Results:**

ST analysis revealed significant shifts in cell composition associated with thyroid cancer dedifferentiation. Specifically, there was an increase in myeloid cells and cancer-associated fibroblasts (CAFs), accompanied by a reduction in natural killer (NK) cells and endothelial cells. Intratumoral heterogeneity in the Thyroid Differentiation Score (TDS) showed a strong spatial correlation with myeloid cell density, and among myeloid subtypes, the MDSC score was negatively correlated with TDS. Pathway activity analysis further identified upregulation of the JAK-STAT and VEGF signaling pathways in dedifferentiated tumors, both of which were closely associated with increased myeloid cell infiltration. IHC validation confirmed the differential expression patterns of representative immune and stromal markers across subtypes.

**Conclusions:**

These findings suggest that dedifferentiation in thyroid cancer is associated with an immunosuppressive TME that promotes tumor progression. Therapeutic strategies targeting JAK-STAT and VEGF pathways, or enhancing NK cell activity, may hold promises for aggressive thyroid cancers. Further studies with larger cohorts are necessary to validate these findings and develop targeted therapies for aggressive thyroid cancers.

**Supplementary Information:**

The online version contains supplementary material available at 10.1007/s00262-025-04210-0.

## Background

Thyroid cancer is the most common endocrine malignancy, with its incidence rising annually until the mid-2010s [1–3]. This increase has largely been driven by the growing prevalence of differentiated thyroid cancers (DTC), including papillary thyroid carcinoma (PTC) and follicular thyroid carcinoma (FTC) [3]. However, recent reports indicate a decline in overall thyroid cancer diagnoses, attributed to changes in diagnostic criteria for early-stage cancers. Despite this trend, the incidence of advanced thyroid cancers continues to rise [2, 4].

Unlike DTC, which generally has an excellent prognosis, poorly differentiated thyroid cancer (PDTC) and anaplastic thyroid cancer (ATC) exhibit markedly worse outcomes, with patients with ATC having a median survival of less than 6 months [5–7]. Efforts to understand these aggressive cancers are increasingly focused on the molecular and genetic changes that drive the dedifferentiation process from DTC to ATC [8, 9].

Treatment of dedifferentiated thyroid cancers involves multimodal approaches, including surgery, radioactive iodine therapy, external beam radiation, and targeted chemotherapy. However, many cases demonstrate poor responsiveness to these standard therapies [10]. This highlights the urgent need for a deeper understanding of the tumor microenvironment (TME) and the exploration of innovative therapeutic strategies, such as immunotherapy.

Given these challenges, this study aimed to analyze the TME of thyroid cancers, including PTC, FTC, PDTC, and ATC, to uncover new insights into their biological behavior. Using spatial transcriptomics (ST) analysis, we focused on the TME within tumor regions and gained further understanding through molecular pathway analysis and correlation studies. To validate and complement these findings, we additionally performed immunohistochemistry (IHC) assays targeting specific TME components.

## Materials and methods

### Sample collection

All samples were obtained from a single specialized cancer institution. A total of 12 patient samples were analyzed, of which three were ATC and five were PDTC; two PTC and two FTC were selected as DTC. All five cases of PDTC occurred against a background of PTC (Table 1). For each patient, diagnosis, sex, age, and TNM classification were collected, along with data on BRAF immunohistochemical staining, mutation status, and histopathological evidence of thyroiditis.

**Table 1:** Clinicopathological characteristics of the 12 thyroid carcinoma cases

No.	Group	Sex	Age	Diagnosis	TNM classification (AJCC2017)	BRAF V600E IHC/BRAF mutation	Thyroid Inflammation (H&E-based)	DV200 (%)
1	FTC	F	67	FTC, widely invasive	pT1bN0	Negative/Not detected	None	67.28
2	FTC	F	54	FTC, minimally invasive	pT1bNx	NA/Not detected	None	79.67
3	PTC	M	47	PTC, classic type	pT1bNx	Positive/V600 codon	None	57.45
4	PTC	M	61	PTC, classic type	pT1bNx	Positive/V600 Codon	None	60.42
5	PTC-PDTC	F	64	PTC with focal undifferentiated carcinoma component (< 5%)	pT3bN1a	Positive/NA	Hashimoto’s thyroiditis	46.29
6	PTC-PDTC	F	55	PTC with focal undifferentiated component (10%)	pT4aN1a	Positive/Not detected	Hashimoto’s thyroiditis	36.14
7	PTC-PDTC	F	90	PTC with undifferentiated component (20%)	pT4aN1a	Equivocal/Not detected	None	57.38
8	PTC-PDTC	F	57	PTC with focal poorly differentiated component (5%)	pT1bN1a	Positive/Exon 15	Lymphocytic thyroiditis	75.41
9	PTC-PDTC	M	60	PTC, oncocytic variant with focal poorly differentiated component (5%)	pT4aN1a	Positive/V600 codon	None	73.96
10	ATC	F	78	Undifferentiated carcinoma	pT4aN0	NA/Not detected	None	47.92
11	ATC	M	91	Undifferentiated carcinoma	pT4aN0	NA/Not detected	None	48.03
12	ATC	F	78	Undifferentiated carcinoma	pT4aN0	Negative/Not detected	None	68.17

IHC, Immunohistochemistry; H&E, Hematoxylin and eosin; FTC, Follicular thyroid carcinoma; PTC, Papillary thyroid carcinoma; PDTC, Poorly differentiated thyroid carcinoma; ATC, Anaplastic carcinoma

### Spatial transcriptome profiling

The regions subject to ST analysis were selected based on a 6.5 × 6.5-mm square area for each case, which was determined by discussion based on hematoxylin and eosin (H&E)-stained images, between the clinicians (J.S. and Y.S.J.) and the experienced pathologist (S.Y.P.) who prepared all H&E slides. All areas were located at the periphery of the tumor, rather than at the center, to analyze the tumor microenvironment. In particular, for the five PDTC cases with a background of PTC, the PDTC areas were included.

RNA was extracted from the selected areas by cutting formalin-fixed paraffin-embedded (FFPE) tissue blocks, and all samples met the RNA quality threshold (DV200 > 30%). Tissue sections were processed using the Visium CytAssist Spatial Gene for the FFPE assay, including library construction and quality control. Libraries were sequenced using the Illumina HiSeq X Ten sequencing system (Illumina, Inc., San Diego, CA, USA**)**. SpaceRanger (v2.0.0) count pipeline was used with GRCh38 transcriptome data from 10 × Genomics to process the FASTQ files.

### Postprocessing for data analysis

Spatial transcriptome data processing was conducted using the Scanpy (v1.9.3) pipeline in Python (v3.9.17) [11]. To remove low-quality spots, spots with fewer than 200 detected genes and those with more than 20% of mitochondrial gene content were excluded. Log-normalization was performed using the ‘*sc.pp.normalize_total*’ function of Scanpy library, ensuring each spot had a total of 10,000 transcript counts.

### Immunohistochemistry assays

IHC was performed on 4 µm sections of FFPE tissue. Slides were deparaffinized, rehydrated, and subjected to antigen retrieval using EDTA buffer (pH 8.0). Heat-induced epitope retrieval was carried out in a microwave oven for 20–40 min, followed by cooling to room temperature and rinsing in distilled water.

For the NK/T cell assay, sections were stained with a rabbit monoclonal anti-CD56 primary antibody and a matched rabbit immunoglobulin G (IgG)-negative control, using the UltraView Universal DAB IHC Detection Kit on the BenchMark ULTRA Plus automated platform. For the detection of endothelial cells, M1/M2 macrophages, cancer-associated fibroblasts (CAFs), and myeloid cells, sections were stained with ERG, CD68, C-MYC, SMA, and CD45 mouse monoclonal primary antibodies, respectively, along with matched mouse IgG-negative controls, using the same detection kit and platform. A detailed list of the primary antibodies used in this study is provided in Table S1 in the supplement.

### Immunohistochemical evaluation

Immunohistochemical slides were evaluated in a blinded manner without access to clinical or pathological information. All cases were scored by a pathologist (S.Y.P.) using the histochemical score (H-score), calculated as the product of staining intensity (graded 0–3) and the percentage of positively stained cells, yielding a score between 0 and 300. Staining intensity was graded on a scale from 0 to 3: 0, no staining; 1, very weak or equivocal staining; 2, definite but mild to moderate staining; and 3, strong and unequivocal staining. Only target cells exhibiting staining in the appropriate nuclear or cytoplasmic/membranous compartment were considered. Nuclear staining was evaluated for ERG, and cytoplasmic or membranous staining for C-MYC, CD56, CD68, SMA, and CD45.

### Analytic algorithms and methods

In spatial transcriptomics, a total of 18,085 genomic data points were obtained from approximately 4000 spots (ranging from a minimum of 3910 to a maximum of 4908) per specimen. Multiple cells could be included in a single spot. Therefore, it was necessary to estimate the relative proportions of different cell types contributing to each spot. The CellDART algorithm estimates the proportions of cell types forming each spot using the reference scRNA-seq data through Adversarial Discriminative Domain Adaptation [12]. The scRNA-seq dataset used as a reference in this study is GSE193581 [13]. The CellDART algorithm was performed with the num_markers value set to 20.

To determine whether each spot was cancerous, the CopyKAT algorithm was used to estimate copy number variation (CNV) [14]. Spots identified as aneuploid by CopyKAT were classified as cancerous regions. Default settings were used for the algorithm options.

The decoupleR package was used to conduct statistical testing of multiple molecular activities [15]. For the PROGENy analysis [16], a Multivariate Linear Model was employed, while Over-Representation Analysis was performed for the REACTOME pathway, ERK pathway, and thyroid differentiation score (TDS) analyses. Differential expression analysis was carried out using Loupe software (v.7.0.0, 10X Genomics, Pleasanton, CA, USA).

To assess the distribution of specific myeloid cell subtypes, we analyzed gene signature scores associated with macrophage polarization (M1 and M2) and myeloid-derived suppressor cells (MDSCs). The M1 and M2 signatures were obtained from curated gene sets in the MSigDB database [17], while the MDSC signature was derived from a previously published study [18]. More specifically, the MDSC signature was defined using the following genes: S100A8, S100A9, CD33, ITGAM, CD14, CD15, ARG1, NOS2, and IL10. Gene signature scores were calculated using the *scanpy.tl.score_genes* function, and each score was subsequently normalized by the corresponding ‘myeloid cell density’ estimated via CellDART. This normalization allowed us to evaluate the relative enrichment of each myeloid subtype within the broader myeloid compartment, thereby capturing population shifts across tumor samples.

In H-score evaluation, the kappa statistical test was used to assess variability between assays. A *P*-value of < 0.05 was considered statistically significant. The statistical analyses were performed using SPSS for Windows, version 23 (IBM SPSS Inc., Chicago, IL, USA).

## Results

### Cell type differences in intratumoral regions according thyroid cancer subtypes

**Fig. 1:**
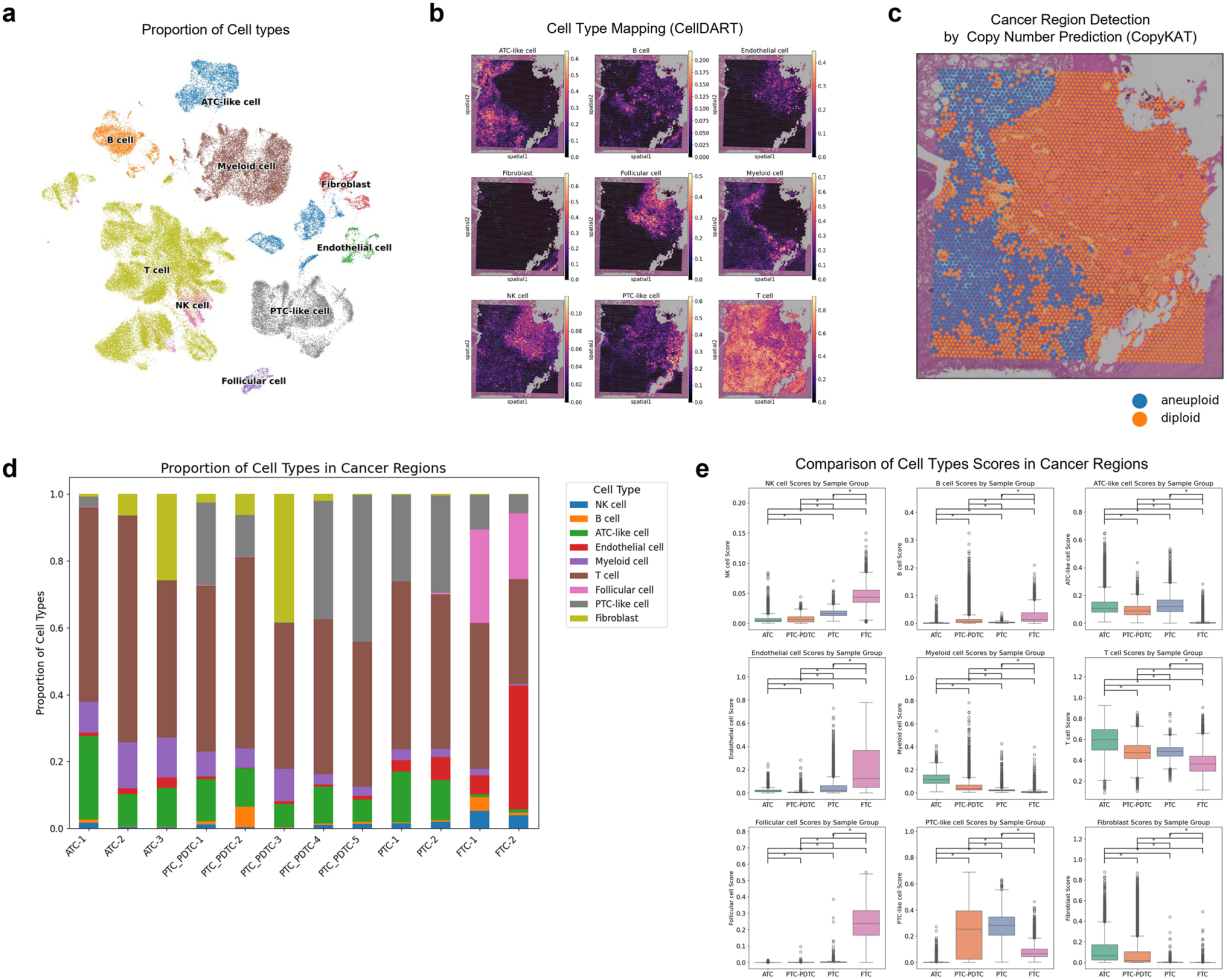
Spatial characterization of tumor microenvironment (TME) dynamics across thyroid cancer differentiation. **a** UMAP visualization of major immune and stromal cell populations inferred from reference scRNA-seq data, showing well-separated clusters. **b** CellDART analysis for delineating TME cell types within tissue sections. **c** Spatial mapping of copy number variation (CNV)-inferred tumor regions overlaid on ST tissue sections. **d** Cell type deconvolution within tumor boundaries, quantifying immune and stromal cell infiltration in cancerous areas. **e** Changes in TME cell composition across thyroid cancer subtypes (ATC, PDTC, and DTC), highlighting increased myeloid cells and fibroblasts, and decreased NK and endothelial cells with tumor progression (*: *p* < 0.05)

To investigate how the TME changes with thyroid cancer differentiation, we performed a spatially resolved analysis of cell type composition using ST data. TME cell types were inferred by deconvoluting the ST data using the reference scRNA-seq dataset, as described in the Methods section. The reference scRNA-seq data enabled identification of major immune and stromal cell populations, which were visualized using UMAP, revealing well-separated clusters corresponding to each cell type (Fig. 1a). To specifically characterize the composition of cancer-infiltrating cells, we first delineated tumor regions within each tissue section. This was achieved by inferring CNVs from spatial transcriptomic data using the CopyKAT algorithm, which allowed for the identification of malignant epithelial regions (Fig. 1b, c). Following this spatial delineation of cancer regions, cell type deconvolution was performed within the tumor boundaries to quantify the relative abundance of immune and stromal cell types infiltrating the cancerous areas (Fig. 1d). Our analysis revealed distinct changes in cellular composition across different types of thyroid cancers: ATC, PDTC, and DTC (comprising PTC and FTC). As tumors progressed from FTC to ATC, there was a marked increase in myeloid cells and fibroblasts, whereas natural killer (NK) and endothelial cells decreased significantly (Fig. 1e).

### Spatially colocalized TME cell types associated with thyroid cancer differentiation

**Fig. 2:**
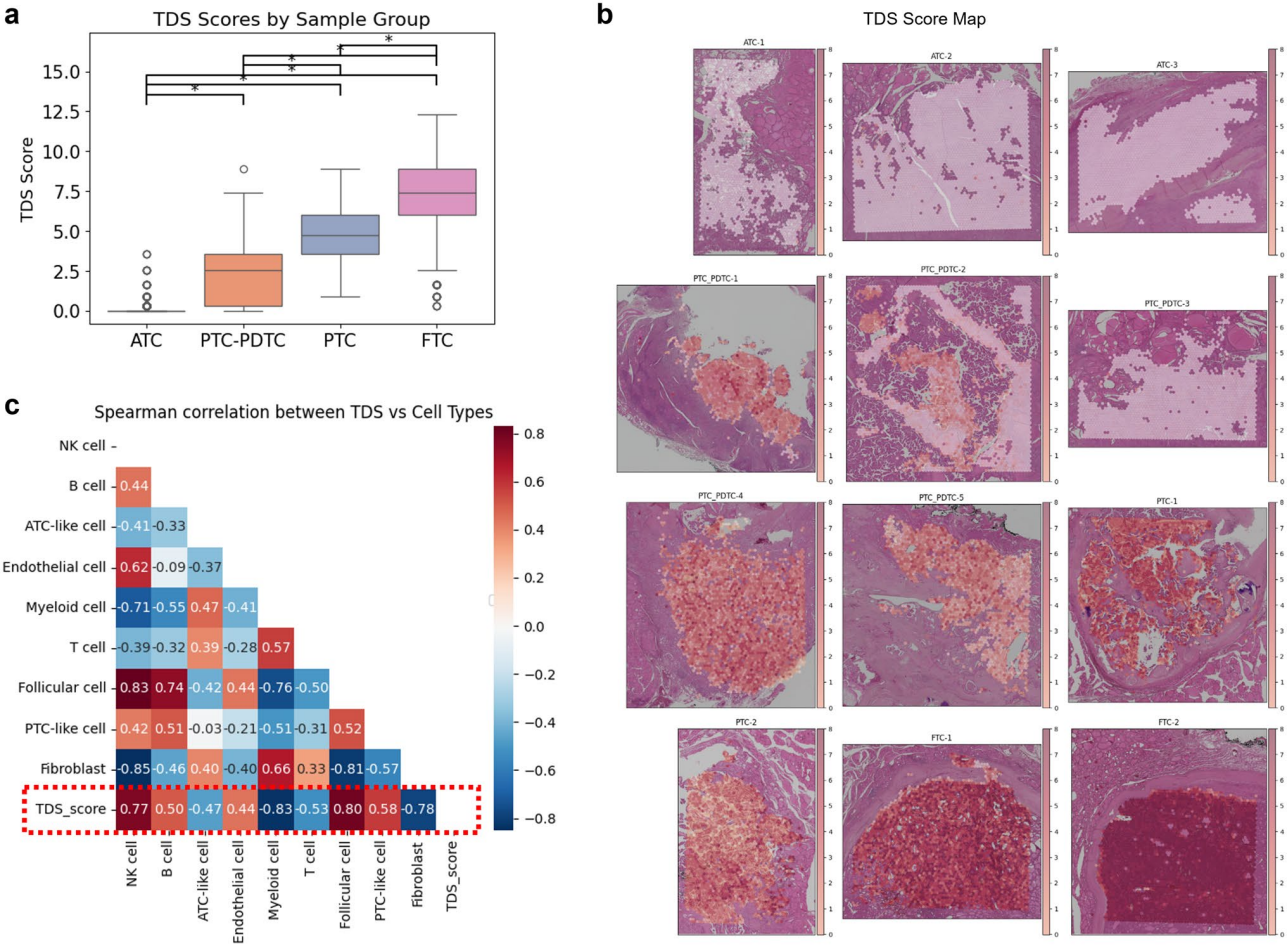
Spatial distribution of Tumor Differentiation Score (TDS) and its correlation with TME cell types. **a** Boxplot of TDS values across thyroid cancer subtypes. **b** Spatial TDS maps overlaid on tissue sections, highlighting regional differentiation patterns. **c** Correlation analysis between TDS and TME cell types densities, revealing inverse associations with fibroblasts and myeloid cells, and a positive correlation with NK cells

**Fig. 3:**
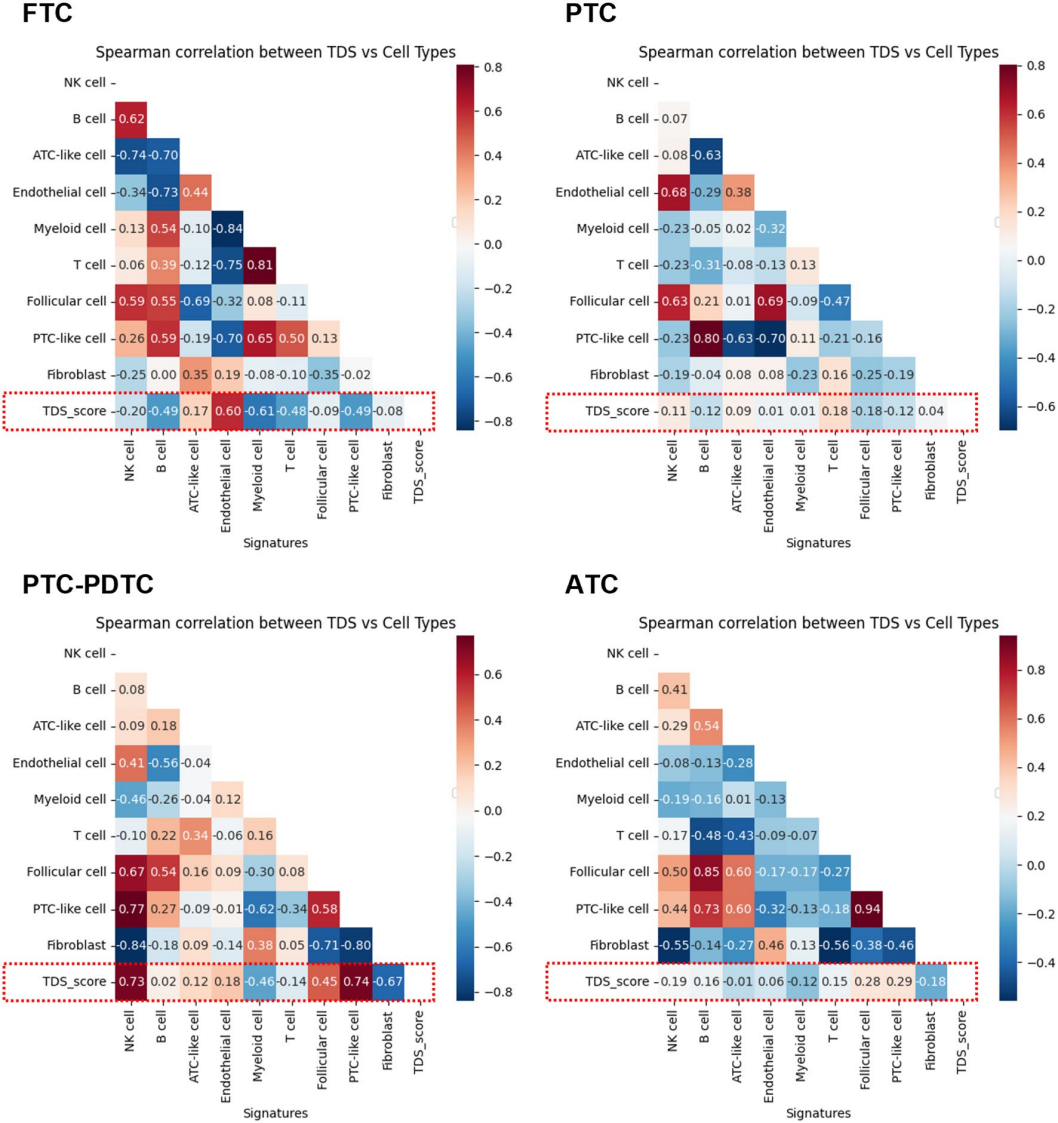
Subtype-specific spatial associations between tumor differentiation and TME cell composition. Correlation plots of TDS with myeloid, fibroblast, NK, and endothelial cell densities across FTC, PTC, PDTC, and ATC. Spatial co-localization patterns showing distinct infiltration dynamics of myeloid and NK cells along the FTC–PTC–PDTC–ATC progression axis

To investigate differentiation-related changes, we calculated the TDS from ST data, computing TDS values for each spot within individual tumors. As expected, TDS varied significantly across histologic subtypes, with FTC exhibiting the highest scores and ATC the lowest (Fig. 2a). Spatial TDS maps overlaid on tissue images (Fig. 2b) further illustrated this pattern, highlighting lower differentiation in ATC regions. Given the spatial heterogeneity of TDS within tumors, we performed correlation analyses with tumor microenvironment (TME) cell types to identify spatial associations between differentiation and immune or stromal components. This analysis revealed an inverse correlation between TDS and the density of fibroblasts and myeloid cells, while NK cell density showed a strong positive association with TDS (Fig. 2c). These trends manifested differently across the various thyroid cancer subtypes—FTC, PTC, PDTC, and ATC. An inverse correlation between TDS and myeloid cell density was consistently observed in PTC, PDTC, and ATC, suggesting that increased myeloid infiltration is a common feature of tumor dedifferentiation across these subtypes. In contrast, the positive correlation between TDS and NK cell density was specifically evident in PDTC, indicating a more subtype-specific relationship. These findings suggest that the accumulation of myeloid cells during dedifferentiation occurs at the level of intratumoral heterogeneity, particularly along the PTC–PDTC–ATC progression axis (Fig. 3). More specifically, in PTC, the association between tumor differentiation and cell infiltration was weaker. In FTC, a decline in endothelial cells correlated with a decrease in differentiation, indicating that endothelial depletion is linked to poorer differentiation. In mixed PDTC tissues, NK cell density decreased as the TDS declined, indicating that NK cell infiltration is reduced in less differentiated tumor regions. Notably, the co-localization of NK cells with differentiation status emerged as a unique pattern and not previously identified.

**Fig. 4:**
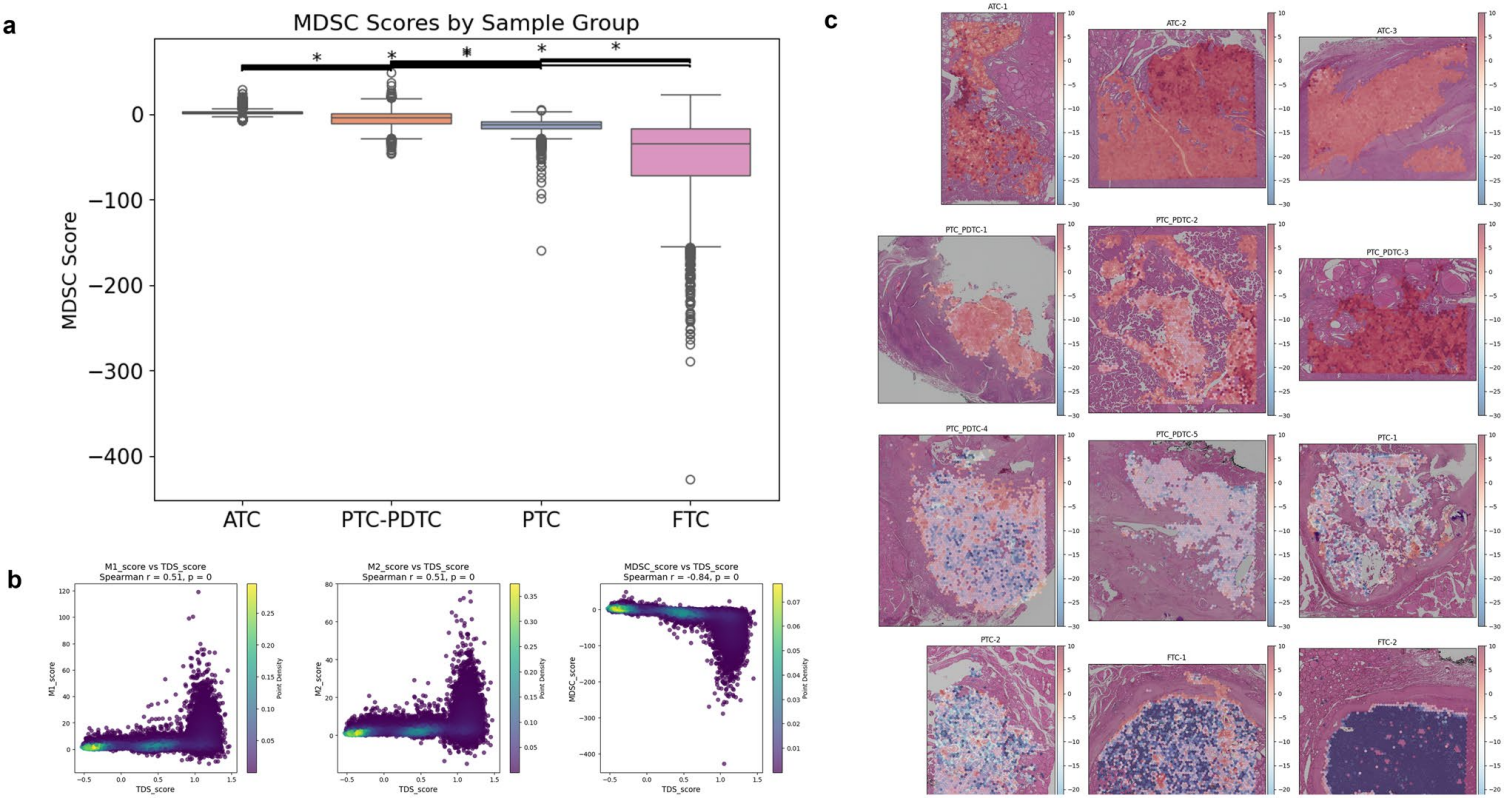
Spatial analysis of myeloid subpopulations and their association with tumor differentiation. **a** Correlation between normalized MDSC scores and TDS across tumor sections, showing a strong inverse relationship. M1 and M2 signature scores were positively correlated with TDS. **b** Boxplots of normalized MDSC scores across thyroid cancer subtypes, with highest enrichment in ATC. **c** Spatial mapping of MDSC signature scores over tissue sections, visualizing the spatial distribution of immunosuppressive myeloid cells within tumor regions

Given the consistent association between myeloid cell density and TDS across tumor types as well as within individual tumors, we further analyzed specific myeloid subpopulations. To assess the relative contribution of different myeloid subsets, we calculated normalized M1, M2, and MDSC scores by dividing their respective gene signature scores by the overall myeloid cell density, thereby reflecting their proportional enrichment within the TME. Among these, the normalized MDSC score showed a strong negative correlation with TDS (*r* = − 0.84, *p* < 0.001; Fig. 4a), with the highest levels observed in ATC and the lowest in FTC (Fig. 4b). This spatial relationship was further confirmed in tissue sections, where normalized MDSC signature scores were mapped across tumor regions (Fig. 4c).

### Immunohistochemical confirmation of spatial transcriptomic results

**Fig. 5:**
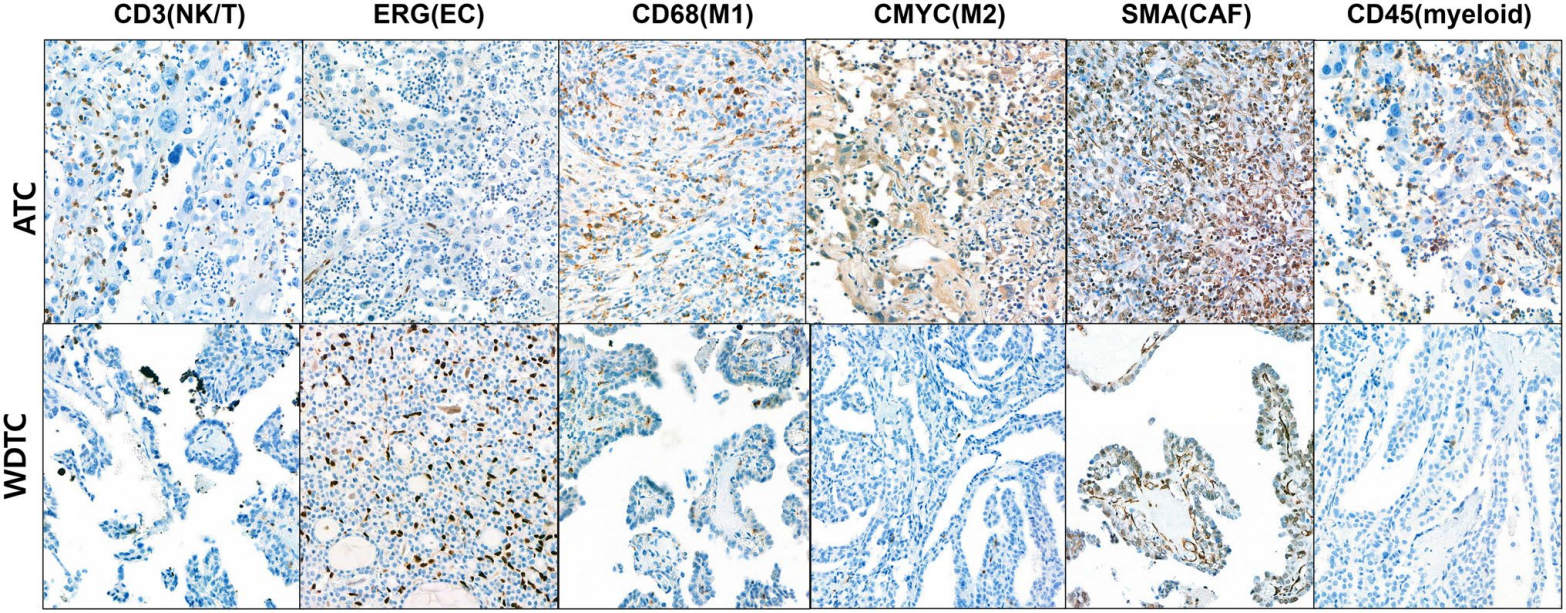
Representative immunohistochemical staining for key TME markers—CD56 (natural killer/T cells [NK/T]), ERG (endothelial cells [EC]), CD68 (M1 macrophages), C-MYC (M2 macrophages), SMA (fibroblasts), and CD45 (myeloid cells)—in representative cases of well-differentiated thyroid cancer (WDTC) and anaplastic thyroid carcinoma (ATC). Scale bars, 100 μm

To validate the cell type composition inferred from ST data, we performed IHC staining for representative immune and stromal markers across a spectrum of thyroid cancer tissues, including FTC, PTC, PDTC, and ATC (Tables 2 and S2 in the supplement). Representative images of positive staining are illustrated in Fig. 5.

**Table 2:** Summary of H-scores for immunohistochemical markers across thyroid cancer cases

No.	Group	CD56/CD3 (NK/T cell)	ERG (Endothelial cell)	CD68 (M1)	C-MYC (M2)	SMA (CAF)	CD45 (Myeloid cell)
1	FTC	0	57	10	10	15	20
2	FTC	0	90	10	2	15	16
3	PTC	0	180	10	20	30	4
4	PTC	0	105	10	6	15	2
5	PTC-PDTC	14	105	60	40	120	120
6	PTC-PDTC	10	45	52	60	225	90
7	PTC-PDTC	30	45	90	50	240	80
8	PTC-PDTC	10	63	90	40	60	60
9	PTC-PDTC	0	33	6	45	30	6
10	ATC	16	30	120	60	240	80
11	ATC	20	54	150	80	240	80
12	ATC	50	60	120	150	240	50

M1, M1 macrophage; M2, M2 macrophage; CAF, Cancer-associated fibroblast; FTC, Follicular thyroid carcinoma; PTC, Papillary thyroid carcinoma; PDTC, Poorly differentiated thyroid carcinoma; ATC, Anaplastic carcinoma

H-scores for fibroblasts (SMA) and myeloid cells (CD45) were low in FTC and PTC, but increased substantially in PDTC and ATC, consistent with the observed accumulation of stromal and immune cells during tumor dedifferentiation.

Endothelial cell staining (ERG) showed the opposite trend, with strong positivity in differentiated tumors and reduced expression in PDTC and ATC, reflecting endothelial loss in poorly differentiated cancers. Similarly, M1 (CD68) and M2 (C-MYC) macrophage markers were minimally expressed in FTC/PTC but increased in PDTC and ATC, in line with ST-inferred enrichment of immunosuppressive myeloid populations.

NK cell staining (CD56) was absent in most FTC and PTC cases, while modest positivity was observed in a subset of PDTC and ATC samples. Although this pattern did not show clear progression, it may reflect subtype-specific variation in NK cell infiltration, partially aligning with spatial transcriptomic trends.

### Pathway activity and its association with differentiation and myeloid cell infiltration

**Fig. 6:**
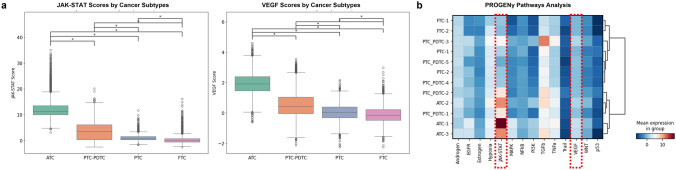
Pathway activity dynamics across thyroid cancer differentiation. **a** PROGENy-inferred activity scores for JAK-STAT and VEGF pathways across FTC, PTC, PDTC, and ATC, showing progressive activation in dedifferentiated tumors. **b** Heatmap visualization of JAK-STAT pathway activity, highlighting regions of high activation in poorly differentiated ATC tissues

Using PROGENy, we calculated the activity scores for key signaling pathways. Among the pathways analyzed, JAK-STAT and VEGF pathway activities showed a significant trend of progressive increase from FTC to ATC, with the highest activity observed in ATC (Fig. 6a). Notably, the JAK-STAT pathway demonstrated the most dramatic increase in expression in the most dedifferentiated tumors (Fig. 6b).

**Fig. 7:**
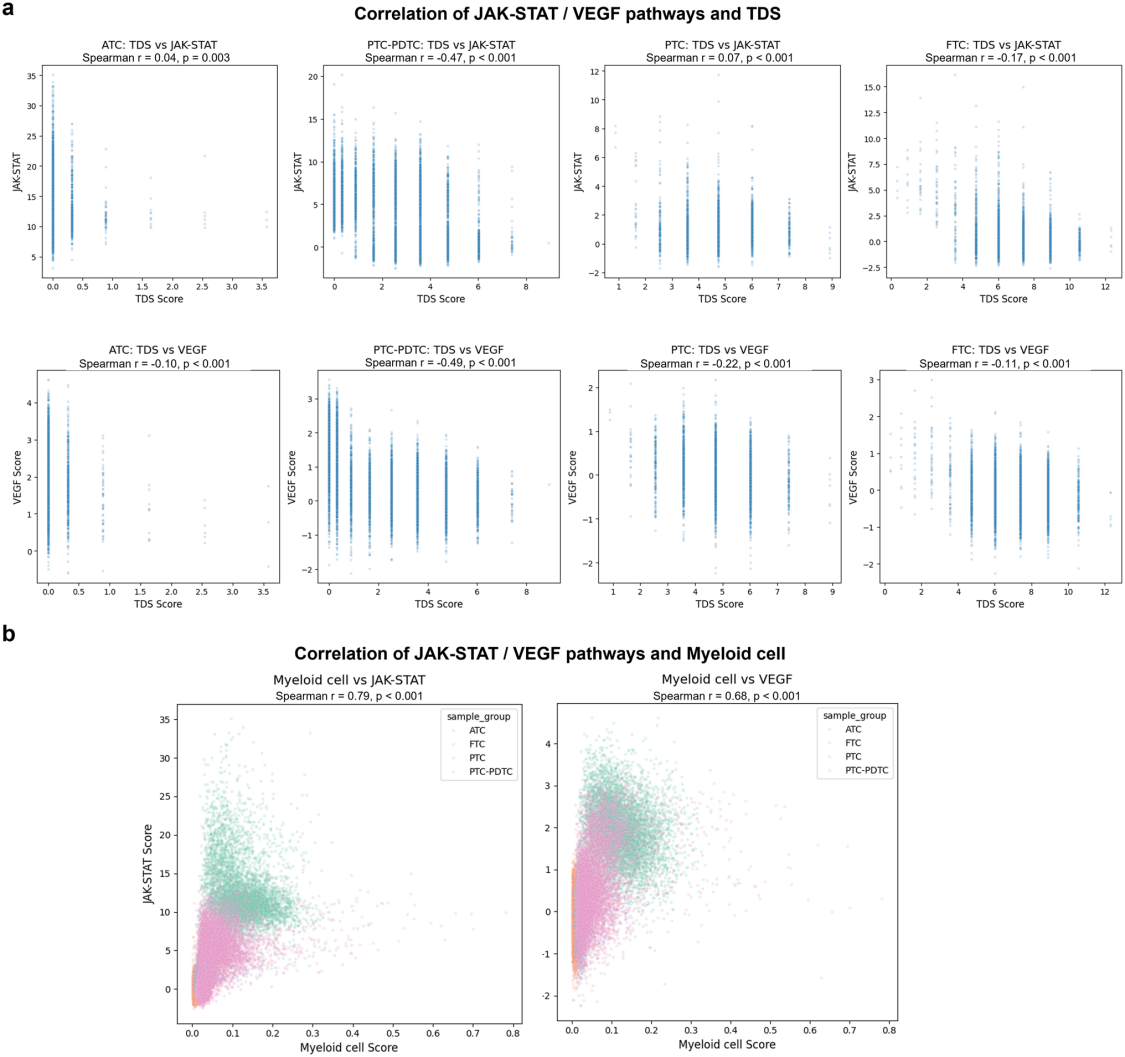
Correlation of pathway activities with tumor differentiation and myeloid cell infiltration. **a** Correlation plots of VEGF and JAK-STAT pathway activities with TDS across tumor subtypes, showing subtype-specific trends. **b** Spearman correlation analysis between pathway activities and myeloid cell infiltration densities, indicating strong associations particularly in PTC-PDTC transition zones

To further investigate the relationship between these pathways and tumor differentiation, we examined their correlation with TDS (Fig. 7). VEGF activity showed a consistent negative correlation with TDS across all tumor subtypes (ATC: Spearman *r* = − 0.10, PTC-PDTC: *r*= − 0.49, PTC: *r*= − 0.22, and FTC: r *r* − 0.11; all *p*< 0.01). In contrast, the JAK-STAT pathway displayed subtype-specific correlations, with a strong negative correlation in the PTC-PDTC group (Spearman *r* = − 0.47), but weaker or inconsistent correlations in other subtypes (ATC: *r* = 0.04, PTC: *r* = 0.07, and FTC: *r* = − 0.17; all *p* < 0.01). Both pathways showed the strongest correlation with TDS in the PTC-PDTC group.

Additionally, when assessing myeloid cell infiltration, both pathways demonstrated a strong association. The JAK-STAT pathway exhibited a Spearman correlation of *r* = 0.79, and VEGF had r = 0.68 (both *p* < 0.001). Examining the relationship between dedifferentiated cancer types and myeloid cell infiltration (Fig. 1e), we found that JAK-STAT pathway activity becomes particularly active during the late stages of dedifferentiation.

## Discussion

Recent advances in molecular genetics have profoundly enhanced our understanding of genetic mutations and alterations in signaling pathways driving thyroid cancer. These advancements have provided deeper insights into the clinicopathological characteristics and prognosis of thyroid cancers, as well as the genetic alterations that contribute to tumor progression. ST is one of the advances in molecular biology which has emerged as a powerful tool for analyzing the complex interactions within the TME [19].

In this study, we used ST to identify distinct differences in cell type composition and pathway activity across thyroid cancer subtypes. Our findings revealed a progressive increase in myeloid cells and fibroblasts, coupled with a significant decline in NK and endothelial cells, as differentiation progressed from DTC to ATC. Of note, endothelial cell decrease supports vascular depletion likely reflects hypoxia and poor perfusion in dedifferentiated tumors, which may contribute to reduced immune infiltration, including NK cells. These trends suggest that dedifferentiation is associated with profound changes in the TME, favoring an immunosuppressive and tumor-promoting microenvironment.

### Myeloid cell infiltration and thyroid differentiation

Myeloid cells, particularly tumor-associated macrophages (TAMs), play a critical role in the thyroid cancer microenvironment. They promote tumor invasion, metastasis [20], and immune evasion by fostering an immunosuppressive environment [21]. Increased TAM density has been associated with advanced stages and poor survival outcomes [22]. In our study, we observed a strong negative correlation between myeloid cell density and TDS, suggesting that myeloid infiltration intensifies as tumor differentiation decreases.

In addition, MDSCs, a heterogeneous population of immature myeloid cells, are key mediators of immunosuppression within the tumor microenvironment. They inhibit T-cell and NK cell activity, promote angiogenesis and metastasis, and contribute to immune evasion and resistance to therapy [18, 23]. In our analysis, MDSCs were found to be relatively enriched within the myeloid cell compartment during tumor dedifferentiation. This increase supports the notion that the expansion of specific myeloid subtypes, particularly MDSCs, is associated with enhanced immune evasion mechanisms in poorly differentiated thyroid cancers.

Both the JAK-STAT and VEGF pathway activities were strongly associated with myeloid cell infiltration, reinforcing their potential role in shaping aggressive TME. These findings highlight the potential of anti-VEGF and anti-JAK-STAT therapies as viable treatment options for poorly differentiated thyroid cancers. Given their roles in immune suppression and angiogenesis, further preclinical and clinical studies are needed to evaluate the therapeutic efficacy of these pathways, particularly for combination strategies.

### Cancer-associated fibroblasts and their role in dedifferentiation

CAFs are another key component of the thyroid cancer TME and contribute to tumor progression through their dynamic interactions with cancer cells. In this study, we observed that CAFs increased progressively from DTC to ATC, supporting the hypothesis that they facilitated dedifferentiation. CAFs undergo tumor-driven reprogramming, secreting factors such as IL-6, ROS, and PDGF that promote tumor proliferation, invasion, and epithelial-to-mesenchymal transition (EMT), while remodeling the extracellular matrix to support malignancy [24]. Given their involvement in aggressive tumor phenotypes, targeting CAF-mediated signaling pathways may represent a promising therapeutic approach for dedifferentiated thyroid cancers.

### NK cells and hypoxia in the dedifferentiated tumor microenvironment

NK cells play a protective role in thyroid cancer by coordinating innate and adaptive immune responses and targeting tumor cells [25]. Our study revealed a strong positive correlation between NK cell density and TDS, indicating that NK cells are more prevalent in well-differentiated tumors. Additionally, hypoxia negatively correlated with NK cell density, highlighting how oxygen deprivation impairs NK cell function. As hypoxia worsens in dedifferentiated tumors, NK cell activity declines, contributing to immunosuppressive TME [26]. However, recent studies suggest that boosting NK cell activation could be a viable therapeutic strategy for dedifferentiated thyroid cancers [27], supporting the need for further research into NK cell-based immunotherapies.

### VEGF pathway activation and endothelial cell dynamics

The reduction in endothelial cells (ECs) observed in this study must be interpreted in the context of increased VEGF signaling pathway activity from DTC to ATC. While ECs typically promote angiogenesis, their decrease in this study suggests alternative mechanisms such as vascular mimicry (VM), where vessel-like structures form without EC involvement [28]. ATC tumors, despite rapid growth, seem primarily supported by enhanced angiogenesis rather than by a proportionate increase in ECs. The potential use of VEGF inhibitors in such tumors increases the complexity and warrants further investigation [29].

### JAK-STAT pathway activation and its clinical implications

The Janus kinase (JAK)-signal transducer and activators of transcription (STAT) pathway represents a promising target for advanced thyroid cancers, given its involvement in tumor proliferation, metastasis, and immune evasion in solid tumors [30]. Its significant upregulation in more aggressive thyroid cancers aligns with these roles, particularly in the context of drug resistance in BRAF^V600E^-positive thyroid carcinoma [31]. In our study, JAK-STAT activation was strongly correlated with myeloid cell infiltration across tumor subtypes and with declining TDS, highlighting its involvement in immune suppression and tumor dedifferentiation. Targeting the JAK-STAT pathway may provide a promising therapeutic approach for dedifferentiated thyroid cancers.

### Potential therapeutic strategies and future directions

Based on our findings, combination immunotherapy may prove beneficial for treating dedifferentiated thyroid cancers. Strategies to enhance NK cell activation, reprogram TAMs, and inhibit JAK-STAT signaling should be explored to improve patient outcomes [20, 32]. Additionally, addressing tumor hypoxia with HIF-1α inhibitors and combining them with VEGF inhibitors could further optimize immune responses. Larger sample sizes and clinical outcome data are needed to validate these therapeutic strategies and establish causal relationships between TME alterations and thyroid cancer progression.

### Limitations

This study has several limitations. The small sample size limits the generalizability of our findings and restricts our ability to establish strong correlations with clinical outcomes. The 12 specimens analyzed may not fully represent the diversity of thyroid cancer subtypes, and the absence of ATC cases originating from FTC represents another limitation in our analysis. Additionally, spatial transcriptomics has inherent technical limitations, such as resolution constraints and potential biases in cell type deconvolution, which should be considered when interpreting the results. Despite these limitations, our study offers valuable insights into the evolving TME in thyroid cancer and highlights key pathways and immune interactions that could be targeted for future therapeutic interventions. Future research should aim to increase the sample size and integrate multi-omics approaches to improve the resolution of cell type deconvolution and pathway interactions.

## Conclusions

This study provides valuable insights into the cellular and molecular dynamics of thyroid cancer progression through ST. Key findings include shifts in cell type composition and pathway activity, highlighting the role of myeloid cells, CAFs, and NK cells in shaping the TME. These findings emphasize the potential for targeting pathways such as VEGF and JAK-STAT, as well as enhancing NK cell activity, as therapeutic strategies for advanced thyroid cancers. To strengthen our results, we performed IHC validation of representative TME markers, which confirmed the ST findings. Expanding analyses to include larger and more diverse cohorts will be crucial to translate these insights into effective therapeutic approaches for patients with dedifferentiated thyroid cancers.

## Supplementary Information

Below is the link to the electronic supplementary material.

Supplementary file1 (DOCX 24 KB)

## Data Availability

The data that support the findings of this study are available on request from the corresponding author.

## Abbreviations

ATC: Anaplastic thyroid cancer
CAF: Cancer-associated fibroblast
CNV: Copy number variation
DTC: Differentiated thyroid cancer
EC: Endothelial cell
EMT: Epithelial-to-mesenchymal transition
ERK: Extracellular signal-regulated kinase
FFPE: Formalin-fixed paraffin-embedded
FTC: Follicular thyroid carcinoma
H&E: Hematoxylin and eosin
HIF-1α: Hypoxia-inducible factor 1-alpha
IgG: Immunoglobulin G
IL-6: Interleukin 6
IRB: Institutional review board
JAK: Janus kinase
NK: Natural killer (cell)
PDGF: Platelet-derived growth factor
PDTC: Poorly differentiated thyroid cancer
PTC: Papillary thyroid carcinoma
ROS: Reactive oxygen species
scRNA-seq: Single-cell RNA sequencing
ST: Spatial transcriptomics
STAT: Signal transducer and activator of transcription
TAM: Tumor-associated macrophage
TDS: Thyroid differentiation score
TME: Tumor microenvironment
VEGF: Vascular endothelial growth factor
VM: Vascular mimicry
