# Supplementary material for: Tumor microenvironment remodeling across thyroid cancer differentiation states revealed by spatial transcriptomics
Source: Cancer Immunol Immunother. 2025 Nov 3;74(12):357. doi: 10.1007/s00262-025-04210-0 (PMC12583360; doi:10.1007/s00262-025-04210-0)
Supplement: Supplementary file 1 — Supplementary file1 (DOCX 24 KB) [file 262_2025_4210_MOESM1_ESM.docx]

**Table S1**. List of primary antibodies used for immunohistochemical staining.

|  | **Clone** | **Source** | **Dilution** | **Detection KIT** | **Retrieval Conditions** | **Incubation Time (min)** | **Incubation Temperature** |
| --- | --- | --- | --- | --- | --- | --- | --- |
| **CD56** | MRQ-42 | Cell Marque | R.T.Ux2 | UltraView Universal DAB Detection Kit | CC1, Mild | 40 | 42℃ |
| **ERG** | EPR3864 | abcam | x100 | UltraView Universal DAB Detection Kit | CC1, Mild | 40 | 42℃ |
| **CD68** | 514H12 | VENTANA | R.T.Ux4 | UltraView Universal DAB Detection Kit | CC1, Mild | 32 | 42℃ |
| **C-MYC** | 9E11 | LEICA | x200 | UltraView Universal DAB Detection Kit | Protease14min | 40 | 42℃ |
| **SMA** | 1A4 | DAKO | x100 | UltraView Universal DAB Detection Kit | No | 32 | 42℃ |
| **CD45** | 2B11 and PD7/26 | DAKO | x400 | UltraView Universal DAB Detection Kit | CC1, Mild | 20 | 42℃ |

※ R.T.U: Ready To Use

※ CC1: pH 8.0 EDTA buffer, 100℃

**Table S2.** Detailed immunohistochemical evaluation of thyroid cancer tissues. For each case and marker, intensity level (I, 0–3) and the percentage of cells (%) are presented.

| **No.** | **Group** | **CD56/CD3**  **(NK/T cell)** | | **ERG**  **(Endothelial cell)** | | **CD68**  **(M1)** | | **C-MYC**  **(M2)** | | **SMA**  **(CAF)** | | **CD45**  **(Myeloid cell)** | |
| --- | --- | --- | --- | --- | --- | --- | --- | --- | --- | --- | --- | --- | --- |
|  |  | I | % | I | % | I | % | I | % | I | % | I | % |
| 1 | FTC | NA | 0 | 3 | 19 | 2 | 5 | 2 | 5 | 3 | 5 | 2 | 10 |
| 2 | FTC | NA | 0 | 3 | 30 | 2 | 5 | 2 | 1 | 3 | 5 | 2 | 8 |
| 3 | PTC | NA | 0 | 3 | 60 | 2 | 5 | 2 | 10 | 3 | 10 | 2 | 2 |
| 4 | PTC | NA | 0 | 3 | 35 | 2 | 5 | 2 | 3 | 3 | 5 | 2 | 1 |
| 5 | PTC-PDTC | 2 | 7 | 3 | 35 | 2 | 30 | 2 | 20 | 3 | 40 | 3 | 40 |
| 6 | PTC-PDTC | 2 | 5 | 3 | 15 | 2 | 25 | 3 | 20 | 3 | 75 | 2 | 45 |
| 7 | PTC-PDTC | 2 | 15 | 3 | 15 | 3 | 30 | 2 | 25 | 3 | 80 | 2 | 40 |
| 8 | PTC-PDTC | 2 | 5 | 3 | 21 | 3 | 30 | 2 | 20 | 3 | 20 | 3 | 20 |
| 9 | PTC-PDTC | NA | 0 | 3 | 11 | 2 | 3 | 3 | 15 | 3 | 10 | 2 | 3 |
| 10 | ATC | 2 | 8 | 3 | 10 | 3 | 40 | 2 | 30 | 3 | 80 | 2 | 40 |
| 11 | ATC | 2 | 10 | 3 | 18 | 3 | 50 | 2 | 40 | 3 | 80 | 2 | 40 |
| 12 | ATC | 2 | 25 | 3 | 20 | 2 | 60 | 3 | 50 | 3 | 80 | 2 | 25 |

Abbreviations: M1, M1 macrophage; M2, M2 macrophage; CAF, cancer-associated fibroblast; FTC, follicular thyroid carcinoma; PTC, papillary thyroid carcinoma; PDTC, poorly differentiated thyroid carcinoma; ATC, anaplastic carcinoma
